# A Rare Case of Extraction of Ventriculoperitoneal Shunt From the Left Main Bronchus

**DOI:** 10.7759/cureus.51021

**Published:** 2023-12-24

**Authors:** Bothayna Amien, Amer Harky, Amy Hill, Michael Shackcloth, Julius Asante-Siaw

**Affiliations:** 1 Cardiothoracic Surgery, Liverpool Heart and Chest Hospital, Liverpool, GBR; 2 Anaesthesia and Critical Care, Liverpool Heart and Chest Hospital, Liverpool, GBR

**Keywords:** airway perforation, idiopathic intracranial hypertension (iih), ventriculoperitoneal (vp) shunt, ventriculoperitoneal shunt complications, upward migration of ventriculoperitoneal shunt

## Abstract

We present the case of a 40-year-old female who underwent several insertions of ventriculoperitoneal (VP) shunts as a part of the treatment for idiopathic intracranial hypertension (IIH). Several years after the insertion of the last VP shunt, the patient started experiencing shortness of breath (SOB) and cough; after further assessment, it was noted on computed tomography (CT) scan that the VP shunt had migrated into the right lower lobe of the lung and perforated the distal left main bronchus. The shunt was successfully retrieved using bronchoscopy under general anesthesia, after which the patient had a complete resolution of symptoms. Shunt migration is one of the rare complications that can happen years after shunt insertion. Therefore, we present this rare case of shunt migration into the thorax cavity to highlight the presentation of this complication and its successful management.

## Introduction

Ventriculoperitoneal (VP) shunt insertion is one of the common procedures performed in neurosurgery. They are the main treatment stay for hydrocephalus and are also used in the management of idiopathic intracranial hypertension (IIH) for cerebral diversion in order to avoid damage to the optic nerve and brain tissues that could happen as a result of the high intracranial pressure (ICP) [1,2]. Despite being an important surgical intervention, VP shunts can pose several complications, such as shunt blockage, hematoma, infection, malposition, and shunt migration [3].

Shunt migration is one of the distressing complications that can present with vague symptoms, making its diagnosis and management difficult. It can be classified according to the compartment the shunt migrates to, including bowel, breast, genitourinary, and thorax cavities [4]. Symptoms differ mainly depending on the compartment affected; management of this complication is case-specific and is influenced by the patient's presentation, the compartment affected, and the condition of the shunt itself. Therefore, we present this rare case of shunt migration into the thorax cavity, which was successfully managed using bronchoscopy.

## Case presentation

**Figure 1:**
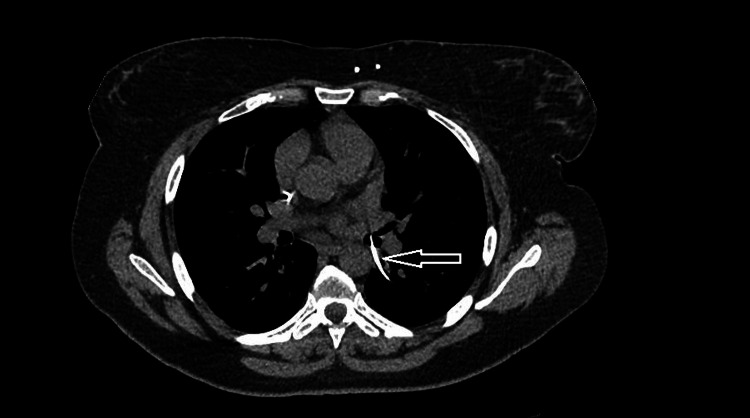
CT scan showing the stent in the right lower lobe CT: computed tomography

A 40-year-old female presented in 2021 with a six-month history of ongoing shortness of breath (SOB), cough, and intermittent wheezing. Her past medical history included IIH, for which she had several VP shunts inserted during her adult lifetime; the last one was inserted in 2013. She was otherwise fit, with no other significant medical history. The patient was referred to our thoracic center due to her ongoing symptoms of SOB and cough. As part of the work-up for her ongoing symptoms, a computed tomography (CT) scan of her thorax showed the redundant stent had migrated into the right lower lobe and perforated the distal left main bronchus (Figure 1). 

The case was discussed at the thoracic surgery multi-disciplinary meeting, and it was decided to list the patient for an initial bronchoscopy assessment of the airways. The patient underwent a rigid bronchoscopy for the assessment of the shunt. During the procedure, the tip of the shunt was visible through the left main bronchus, and an attempt was made to remove the shunt using rigid bronchoscopy forceps; the shunt was pulled easily, and the full shunt was successfully extracted during the procedure (Video 1).

**Video 1:** Video taken during bronchoscopy showing shunt retrieval from the left main bronchus

**Figure 2:**
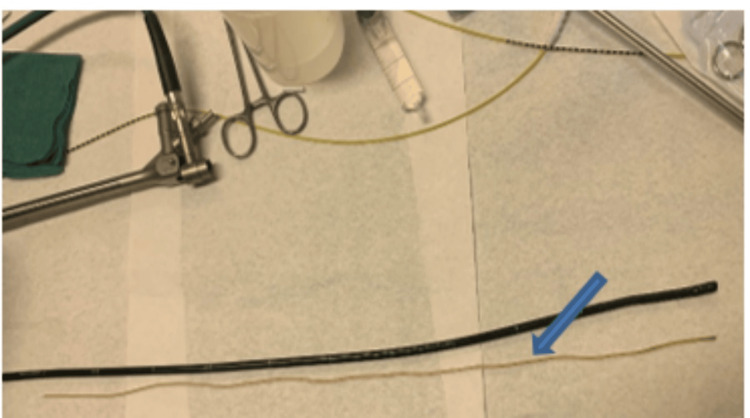
Shunt shown after extraction

After the shunt was removed safely, it wasn't possible to see the perforation in the bronchus during the bronchoscopy (Video 2). The shunt was 55 cm in length when compared to the bronchoscope (Figure 2).

**Video 2:** Video taken during bronchoscopy after shunt removal

**Figure 3:**
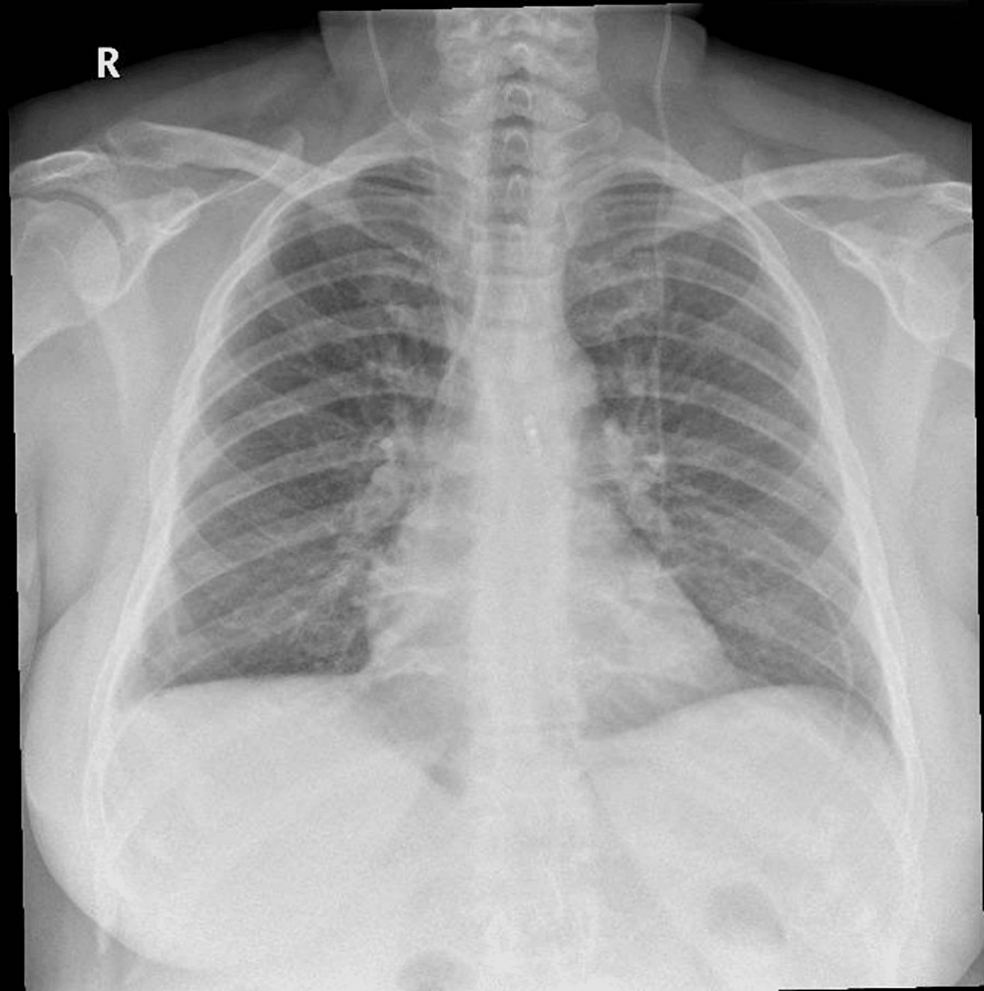
CXR done after shunt removal. Normal lungs and no pneumothorax noted CXR: chest radiography

Postoperatively, chest radiography (CXR) was done to rule out pneumothorax after the procedure. The CXR showed normal lungs with no evidence of pneumothorax (Figure 3). Following the procedure, the patient had a full recovery and was discharged the following day with no complications. The patient presented to the thoracic outpatient clinic four weeks after her procedure as part of a routine follow-up and reported complete resolution of her SOB and cough; the patient was discharged safely from the thoracic clinic.

## Discussion

Different approaches have been utilized for draining the excess cerebrospinal fluid (CSF), including VP shunts. Blockage and infection are the most common causes of failure that require replacement [5]. Although listed in the literature, shunt migration was roughly noted in one of 1000 patients who underwent shunt insertions [4]. It's relatively uncommon as compared to other complications. Shunt migration can be classified according to the site; the most common site is the bowel, followed by the genitourinary system and intracranial [4]. Bowel perforation is a recognized complication, with protrusion of the tip of the catheter through the anus as the most common presentation [6]. Migration into the chest/thorax represents around only 8% of the cases reported in the literature, and 81% of those cases were children [4]. The first case was reported in a 14-month-old child in 1977 by Obrador and Villarejo [7]. Congenital diaphragmatic defects, such as Morgagni and Bochdalek's hernia, are points through which shunts can migrate [4]. The mechanism of thorax migration can be attributed to the positive pressure in the abdomen versus the negative pressure in the thorax, which can favor migration into the thorax. Migration into the thorax can be classified as transdiaphragmatic (through the diaphragm) or supradiaphragmatic (above the diaphragm) [4].

The symptoms are usually acute or chronic respiratory symptoms; however, patients can rarely present with shunt dysfunction [4] but can also present with pneumonia. Corns et al. presented the case of a 50-year-old male with recurrent pneumonia; he had a shunt insertion in 1944, and a CT scan showed the distal end of the shunt within the right-sided bronchus [8]. Other respiratory complications also include pneumothorax, bronchial fistula, hydrothorax, and empyema [8].

Treatment options mainly depend on the patient's presentation. If the patient is presenting with pneumonia or pneumothorax, treatment of the main symptoms is advised, followed by shunt review or assessment [4]. The shunt extraction could be via a surgical or endobronchial method. In our case, the shunt was visible under bronchoscopy. Therefore, endobronchial removal of the shunt from the left main bronchus using rigid bronchoscopy was attempted.

## Conclusions

Shunt migration into the thorax cavity is a rare complication of VP shunt insertion, with very few cases reported in adults. Clinical suspicion is indicated in patients presenting with respiratory symptoms, even several years after shunt insertion. Removal of the shunt is indicated in this complication. In our case, after the shunt migration was identified on CT scan, it was successfully removed using bronchoscopy under general anesthesia, and the patient made a full recovery with complete resolution of symptoms.
